# Evaluation of ESA Active, Passive and Combined Soil Moisture Products Using Upscaled Ground Measurements

**DOI:** 10.3390/s19122718

**Published:** 2019-06-17

**Authors:** Luyao Zhu, Hongquan Wang, Cheng Tong, Wenbin Liu, Benxu Du

**Affiliations:** 1 Institute of Agricultural Remote Sensing and Information Technology Application, College of Environmental and Resource Sciences, Zhejiang University, Hangzhou 310058, China; luyao_zhu@zju.edu.cn (L.Z.); 21714124@zju.edu.cn (C.T.); 2 Key Laboratory of Water Cycle and Related Land Surface Processes, Institute of Geographic Sciences and Natural Resources Research, Chinese Academy of Sciences, Beijing 100101, China; liuwb@igsnrr.ac.cn; 3 Natural Resources Service Center, Dalian 116021, China; dubenxu1116@163.com

**Keywords:** ESA CCI soil moisture, validation, ground measurements upscaling, spatiotemporal analysis

## Abstract

The European Space Agency (ESA) Climate Change Initiative (CCI) project combines multi-sensors at different microwave frequencies to derive three harmonized soil moisture products using active, passive and combined approaches. These long-term soil moisture products assist in understanding the global water and carbon cycles. However, extensive validations are a prerequisite before applying the retrieved soil moisture into climatic or hydrological models. To fulfill this objective, we assess the performances of three CCI soil moisture products (active, passive and combined) with respect to *in-situ* soil moisture networks located in China, Spain and Canada. In order to compensate the scale differences between ground stations and the CCI product’s coarse resolution, we adopted two upscaling approaches of Inverse Distance Weighting (IDW) interpolation and simple Arithmetic Mean (AM). The temporal agreements between the satellite retrieved and ground-measured soil moisture were quantified using the unbiased root mean square error (ubRMSE), RMSE, correlation coefficients (R) and bias. Furthermore, the temporal variability of the CCI soil moisture is interpreted and verified with respect to the Tropical Rainfall Measuring Mission (TRMM) precipitation observations. The results show that the temporal variations of CCI soil moisture agreed with the *in-situ* ground measurements and the precipitation observations over the China and Spain test sites. In contrast, a significant overestimation was observed over the Canada test sites, which may be due to the strong heterogeneity in soil and vegetation characteristics in accordance with the reported poor performance of soil moisture retrieval there. However, despite a retrieval bias, the relatively temporal variation of the CCI soil moisture also followed the ground measurements. For all the three test sites, the soil moisture retrieved from the combined approach outperformed the active-only and passive-only methods, with ubRMSE of 0.034, 0.050, and 0.050–0.054 m^3^/m^3^ over the test sites in China, Spain and Canada, respectively. Thus, the CCI combined soil moisture product is suggested to drive the climatic and hydrological studies.

## 1. Introduction

Soil moisture (SM) is one of the most important land surface parameters, as it has a significant impact on vegetation growth, ecosystems, water cycle, agricultural production and climate change [1]. Thus, monitoring of soil moisture at regional and global scales is essential to deepen our insights into the physical processes of the global water cycle [2], crop growth [3] and drought events [4]. For studying the hydrological [5] and climate change [6] factors, the long-term soil moisture is particularly necessary, which acts as a key driving parameter for the land surface process models. Consequently, soil moisture is considered to be an essential climate variable [7]. 

The traditional soil moisture information was collected via the ground point measurements which are labor- and time-consuming and make it difficult to capture the spatiotemporal variation. In contrast, the remote-sensing techniques provide an efficient tool to estimate soil moisture at varying spatiotemporal scales using the electromagnetic waves. Furthermore, compared to the visible electromagnetic spectrum, the microwaves at long wavelength are less affected by the atmosphere and clouds, allowing us to penetrate vegetation and operate at all-weather conditions [7]. Thus, the microwave signal has become one of the most effective sources to estimate the soil moisture at different spatial and temporal resolutions. The quality of the soil moisture retrieval depends on two factors: the sensor systems and the retrieval algorithms which transform the data into useful geophysical parameters. There are mainly three methods to retrieve the surface soil moisture from microwave data: (i) active radar is based on the inversion of backscattering coefficients, (ii) passive radiometer is based on the inversion of brightness temperature, and (iii) active–passive approaches combine the advantages of radar and radiometer signals. 

The SMOS (Soil Moisture and Ocean Salinity) [2,8] satellite launched in 2009 has contributed to monitor soil moisture and ocean salinity, while the SMAP (Soil Moisture Active Passive) [9,10] satellite launched in 2015 is dedicated to retrieve the soil moisture and soil thaw/freezing status. In particular, the SMAP mission aimed to retrieve soil moisture at a moderate spatial resolution by disaggregating the brightness temperature using the radar backscattering coefficients. Unfortunately, the radar component failed to run after three months’ operation [11]. The independent radar system such as Sentinel-1 [12] was combined with the SMAP radiometer signals for a joint retrieval [13]. Nevertheless, the temporal coverage of these two satellites is rather short, limiting the long-term studies related to climate and hydrology. Since 2010, the European Space Agency Climate Change Initiative (ESA CCI) has integrated active (ERS-1/2 SCAT [14], MetOp ASCAT [15,16]) and passive (SMMR [17], SSM/I [18], TMI [19], AMSR-E [20], WindSat [21], AMSR2 [22] and SMOS [8]) microwave sensors to develop a global long-term (37–39 years) soil moisture product [23,24,25,26,27], which significantly benefits research into global evapotranspiration and climate change.

However, before the application of the ESA CCI soil moisture products, intensive validations are required under different soil, vegetation and climate conditions in order to verify their applicability and uncertainty over different regions [28]. For instance, the CCI products were assessed in Europe [28,29,30], Asia [31,32,33] and Africa [34] using the *in-situ* ground soil moisture observation stations. Nevertheless, it is challenging to match the spatial scales between the CCI soil moisture at coarse resolution and the *in-situ* point measurements. Upscaling methods were needed to compensate the differences in spatial scales, making the inter-comparison possible. The upscaling methods include simple Arithmetic Mean (AM) [35,36], Inverse Distance Weighting (IDW) interpolation, nearest neighbor [28] and kriging interpolation methods [37,38]. The different upscaling methods used soil texture and crop characteristics to account for the spatial autocorrelation and heterogeneity. Considering the efficiency, our study evaluates the two upscaling methods of AM and IDW to process the *in-situ* data within the same pixel, for a comparison with the CCI retrieved soil moisture. 

Actually, different versions of CCI SM products have been released, and our study assesses the performance of version 03.3 using the *in-situ* soil moisture networks located in China, Spain and Canada. At present, CCI SM products have not been verified in Canada, so our study will reveal their performances over high-altitude areas which are significantly influenced by the soil thawing–freezing cycle. Although some validations were conducted in China [31,32,33] and Spain [28,29] before, our study will deepen the understanding of the CCI SM uncertainty using different upscaling and processing approaches. 

## 2. Materials and Methods 

### 2.1. Study Sites and Ground-Based Soil Moisture Measurements

The ground soil moisture measurements were obtained from the International Soil Moisture Network [24] (ISMN: https://ismn.geo.tuwien.ac.at/en/), resulting from an international cooperation to establish and maintain a global ground-based soil moisture database. This database is essential for calibrating, validating and improving remotely sensed soil moisture, and also useful for initiating the land surface, climate, and hydrological models.

**Figure 1:**
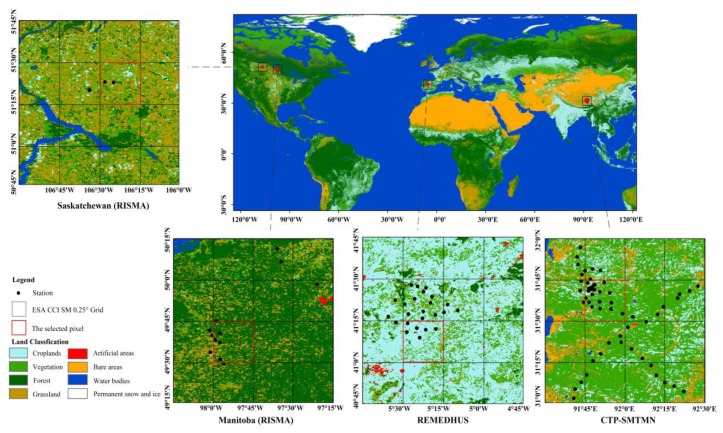
Locations of Climate Change Initiative (CCI) pixels, soil moisture ground measurements and land covers across study areas.

**Figure 2:**
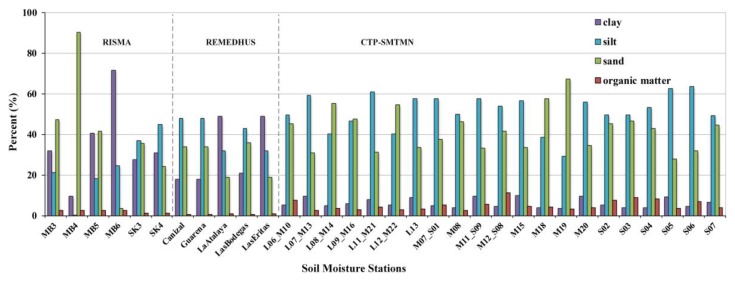
Soil texture (sand, silt, clay) and organic matter over *in-situ* soil moisture stations.

In this study, we selected three probe soil moisture networks located in China, Spain and Canada, as shown in Figure 1. These stations are characterized by different climatic, hydrological, soil and vegetation characteristics, providing three different baselines to evaluate the quality of the CCI remote-sensing soil moisture products. In addition, Figure 2 summaries the information of soil texture (sand, silt, clay) and organic matter over the *in-situ* stations.

The selected soil moisture networks distributed over different areas:

- The Soil Moisture/Temperature Monitoring Network (SMTMN) was established over an area of 100 km × 100 km in the Central Tibetan Plateau (CTP), which is the highest plateau in the world and considered the ‘Third pole’. Over this site, 56 stations were installed to measure soil moisture and temperature at four soil depths (0–5, 10, 20, and 40 cm) and at 30 min temporal interval. Grass is the major vegetation with low biomass, and the variation of soil texture across the plateau leads to a large dynamic of soil moisture [39]. Considering the low penetrating depth of the microwave used to develop the ESA CCI soil moisture product, our study used probe-measured soil moisture at 0–5 cm depth. The probe measurements were calibrated in terms of soil texture as well as soil organic carbon content [40].
- The Red de Estaciones de Medición de la Humedad del Suelo (REMEDHUS) network is located within an area of 30 km × 40 km in the central semiarid of the Duero basin in Spain [41]. It has a semi-arid Mediterranean climate. The land is mainly covered by agricultural crops including cereals and vineyards [41]. This network contains 24 stations equipped with capacitance probes (Stevens Hydra Probe) installed horizontally at a depth of 5 cm.
- The Real-Time In-Situ Soil Monitoring for Agriculture (RISMA) soil moisture network distributes across Canada, with 12 stations in Manitoba, 4 stations in Saskatchewan and 6 stations in Ontario [42]. In this study, we selected Manitoba and Saskatchewan sites for test, they cover an area of around 15 km × 70 km and 10 km × 25 km, respectively. The Ontario site was disregarded, as the CCI pixel covered only a few stations. All stations were designed to record real dielectric permittivity, soil moisture and soil temperature using hydra probes at surface 0–5 cm, 5 cm, 20 cm and 50 cm, while some of these stations reached a deeper depth at 100 cm and 150 cm. At each depth, two or three hydra probe sensors were installed to capture the spatial variability in soil moisture, and to provide alternative measurements in the case of any sensor malfunction. Similarly, we extracted 0–5 cm soil moisture records from this network.

### 2.2. European Space Agency Climate Change Initiative (ESA CCI) Remotely Sensed Soil Moisture Products

In this study, we used the ESA CCI soil moisture products version 03.3 obtained from the ESA data archive (http://www.esa-soilmoisture-cci.org/). These products were generated using active (ERS1-2 SCAT, MetOp ASCAT) and passive (SMMR, SSM/I, TMI, AMSRE, WindSat, AMSR2 and SMOS) microwave space-borne instruments, covering the period from November 1978 to December 2016. The soil moisture was retrieved by three approaches: active-only, passive-only and a combination of them [26,27]. The active method was based on the Soil Water Retrieval Package which estimates the soil saturation degree using the reference backscattering coefficients corresponding to extreme dry and wet conditions. The passive method used the Land Parameter Retrieval Model (LPRM) to extract the soil moisture from brightness temperature [43]. The active and passive retrieved soil moisture were then merged with different weights for a combined product [44]. Through the multi-sensor combination, the CCI product provides global daily surface soil moisture at a spatial resolution of 0.25°.

### 2.3. Spatial Distribution of Global CCI Retrieved Soil Moisture

**Figure 3:**
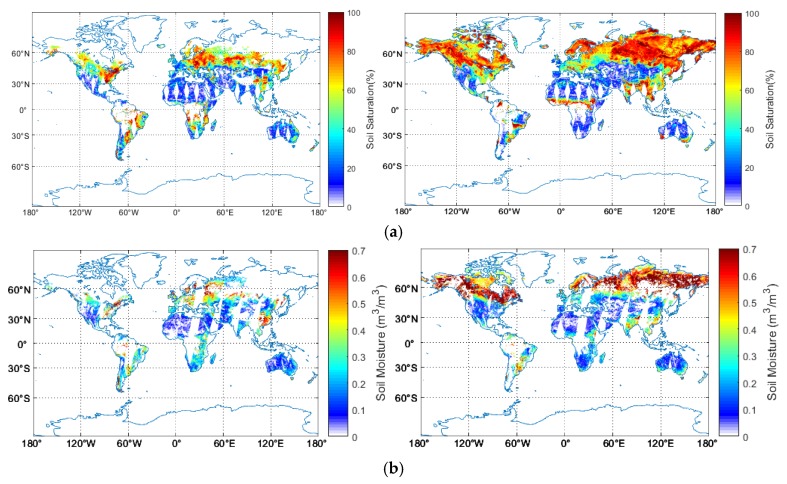

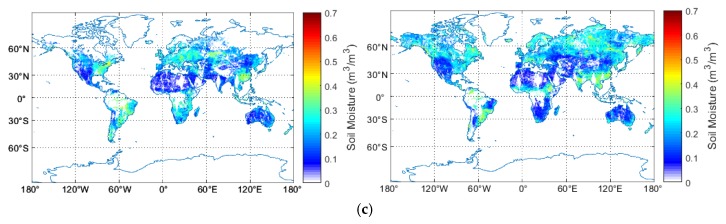
Spatial distribution of European Space Agency Climate Change Initiative (ESA CCI) soil moisture retrieved from (**a**) active (**b**) passive (**c**) combined microwave signals on 15 April (left) and 27 July (right) 2014.

Figure 3 shows the global spatial distribution of the ESA CCI soil moisture from active radar, passive radiometer data and their combinations. As a previous description, due to the retrieval algorithm (by referring to the radar backscattering at extreme wet and dry soil conditions, respectively) used in the active data, the resulting soil moisture is given as saturation index (Figure 3a), by contrast with the standard volumetric soil moisture (Figure 3b,c). Thus, this may prohibit the direct comparison with the passive and combined approaches. Although the conversion from the saturation index to the volumetric soil moisture values was achieved, it may introduce additional bias due to the uncertainty in the required porosity estimates. As the retrievable pixels are different between the active and passive methods, their combination increased the retrieval rate significantly. 

Indeed, the long-term series of ESA CCI soil moisture was realized through multi-sensor combinations. For the year of 2014 in Figure 3, the soil moisture was mainly obtained through the inversion of ASCAT A-B for the active approach, and SMOS and AMSR2 for the passive approach [25]. Consequently, the differences in the sensor configurations also caused retrieval uncertainty. In particular, the different frequency leads to varying penetration depth, resulting in complex interaction between the microwave and the vegetation and soil layers.

In Figure 3, the invalidity pixels may due to several reasons. In the early years, the radiometer often operated at high frequency such as X-, Ku-, K- and Ka- bands. In this case, the microwave penetration depth is rather shallow. Thus, during the vegetated seasons, the backscattering or the emission signals are dominated by the vegetation, limiting the sensitivity of the microwave signals to soil dielectric constants. Furthermore, the retrieval process without convergence may also result in invalid pixels.

### 2.4. TRMM Precipitation 

The Tropical Rainfall Measuring Mission (TRMM) precipitation data were widely validated using the ground gauge observations around the world, while this study used them as a reference to interpret the temporal behavior of CCI soil moisture product. For a given land surface, the soil moisture can be approximated by the differences between the precipitation and evapotranspiration, assuming weak runoff. Thus, the precipitation event may cause the change of soil moisture. In this study, the daily TRMM precipitation products (3B42 V7) given in millimeters at 0.25° × 0.25° spatial resolution were obtained from the National Aeronautics and Space Administration (NASA) GES DISC (https://disc.gsfc.nasa.gov/). The selected spatial resolution is close to the ESA CCI SM product [45]. For a given day, simple summation of the valid retrieved 3-hourly precipitation data in a grid cell was performed to calculate a daily precipitation amount. As described in [46], due to the scale differences between satellite remotely sensed soil moisture (0.25° × 0.25°) and ground point measurements, the inclusion of the precipitation at the same spatial resolution (0.25° × 0.25°) such as TRMM observations is an alternative way to interpret the spatiotemporal distribution patterns of the retrieved soil moisture.

### 2.5. Collocation and Comparison Strategy 

For a given pixel in the ESA CCI SM product, the coordinates of pixel center are given, allowing us to determine that the ground measurements are within or outside the pixel. The distances between the location of a given ground measurement and all the CCI SM pixel centers were calculated, and the CCI SM pixel with the minimum distance is matched to this ground data. After calculation, the 56 stations of the CTP-SMTMN network were found to distribute across 12 pixels of CCI SM. As we focused on the temporal dynamics of soil moisture, only the representative pixels covering the maximum number of ground measurements were selected. The optimal pixels covered 21 stations for the CTP-SMTMN network, five stations for the REMEDHUS network, and six stations for the RISMA network. 

Furthermore, the three ESA CCI soil moisture products were given in different units. The combined and passive products were denoted by volumetric soil moisture (m^3^/m^3^), while the active product is given by saturation degree (%). These different units result from the retrieval algorithms applied to the passive and active microwave data. As per the previous description, the soil moisture from radar data is obtained by referring to the backscattering coefficients in wet and dry soil conditions, leading to a result of saturation degree rather than volumetric soil moisture. In order to conduct the inter-comparison among three soil moisture products (active, passive and combined), a unit conversion from saturation degree to volumetric soil moisture is conducted:

(1) $$SM_{vol}\left(m^{3}/m^{3}\right)=Q\left(\%\right)\times P\left(m^{3}/m^{3}\times 100\%\right)$$

where the *SM_vol_* and *Q* are the volumetric soil moisture and saturation degree, respectively. The porosity *P* was provided by the ESA CCI SM team, it was calculated by accounting the fractions of clay, sand, silt, and organic matter [47]. 

### 2.6. Upscaling Ground Measured Soil Moisture to Match the ESA CCI Products

The ESA CCI SM data at pixel size of 0.25° and the point ground-based soil moisture stations are characterized by significant different spatial scales. A CCI SM pixel (about 600 km²) includes multiple ground-based point measurements [48]. Thus, for each CCI SM pixel, an upscaling of the ground-based station data within the pixel is conducted to obtain a value to represent the ground data corresponding to the pixel. This match between small and large spatial scales allows a comparison between the retrieved and ground measured soil moisture.

In this study, we used two upscaling methods: simple Arithmetic Mean (AM) and Inverse Distance Weighting (IDW) interpolation. The AM calculates the average value of the all the known ground point measurements fallen within the pixel regardless their locations. By contrast, the IDW calculates the potential value corresponding to the pixel center using the known coordinates of the ground point measurements.

(2) $$y(x)=\frac{\sum_{i=1}^{N}w_{i}(x)\cdot y_{i}}{\sum_{i=1}^{N}w_{i}(x)}$$

where the weight of each known soil moisture measurements are given as:

(3) $$w_{i}(x)=\frac{1}{d{\left(x,x_{i}\right)}^{p}}$$

with the distance *d* between the point measurement and the pixel center, and power *p* = 1. 

### 2.7. Statistical Metric for Comparison

To quantify the consistency between CCI SM and upscaling ground SM, four statistical metrics of unbiased root mean square error (*ubRMSE*), *RMSE*, mean difference (bias) and Pearson correlation coefficient (*R*) were computed.

(4) $$ubRMSE=\sqrt{\bar{{\left(\sum_{i=1}^{n}\left(mv_{CCI}(i)-\bar{mv_{CCI}}\right)-\sum_{i=1}^{n}\left(mv_{gd}(i)-\bar{mv_{gd}}\right)\right)}^{2}}}$$

(5) $$RMSE=\sqrt{\bar{{\left(mv_{CCI}-mv_{gd}\right)}^{2}}}$$

(6) $$Bias=\bar{mv_{CCI}-mv_{gd}}$$

(7) $$R=\frac{\sum_{i=1}^{n}\left(mv_{CCI}(i)-\bar{mv_{CCI}})(mv_{gd}(i)-\bar{mv_{gd}}\right)}{\sqrt{\sum_{i=1}^{n}{\left(mv_{CCI}(i)-\bar{mv_{CCI}}\right)}^{2}\cdot \sum_{i=1}^{n}{\left(mv_{gd}(i)-\bar{mv_{gd}}\right)}^{2}}}$$

where *n* is the number of days with both available retrieved and ground measured soil moisture. The *mv_CCI_* and *mv_gd_* are the satellite retrieved and ground soil moisture, respectively. The overbar represents the average process. 

## 3. Results and Discussion

### 3.1. Central Tibetan Plateau Soil Moisture/Temperature Monitoring Network (CTP-SMTMN) 

**Figure 4:**
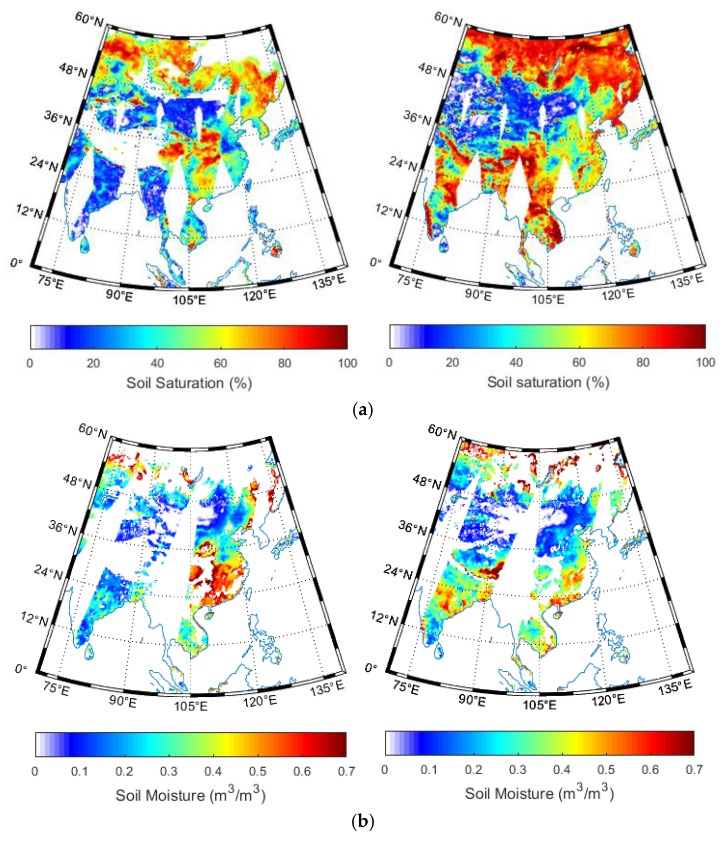

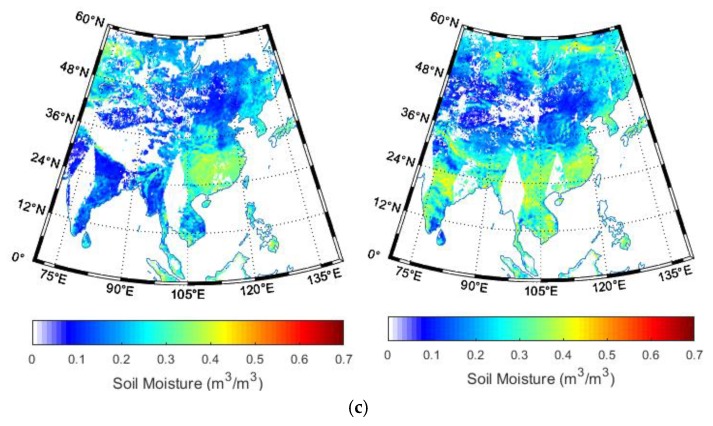
Extracted soil moisture retrieved from (**a**) active (**b**) passive (**c**) combined microwave signals over China on 15 April (left) and 27 July (right) 2014.

**Figure 5:**
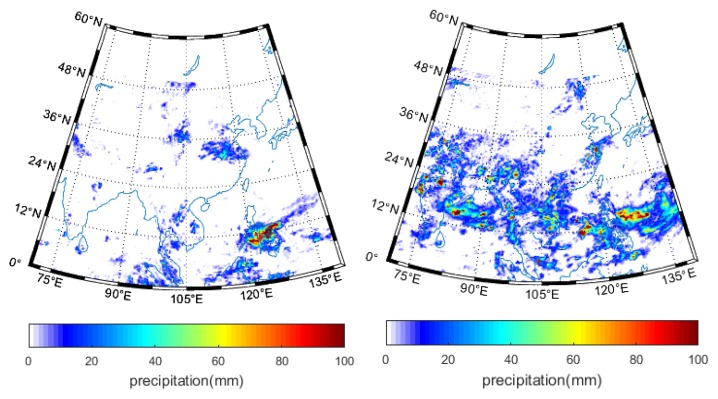
TRMM precipitation over China on 15 April (**left**) and 27 July (**right**) 2014.

We interpreted the CCI soil moisture using both the ground measurements and referring to the precipitation amount. Figure 4 extracts the study area in China in order to verify the details of the soil moisture spatial pattern. The soil moisture in southeast part is significantly higher than in the northwest. This is expected, as the precipitation in southeast China is much stronger in southeast than northwest [49]. Unfortunately, although we extracted the TRMM precipitation to further interpret the CCI soil moisture distribution, most of the TRMM pixels are impossible to retrieve for the selected two days. However, we can still observe the overall higher precipitation over the southeast than northwest in China in Figure 5.

#### 3.1.1. Characteristics of Soil Moisture Evolution over Time

**Figure 6:**
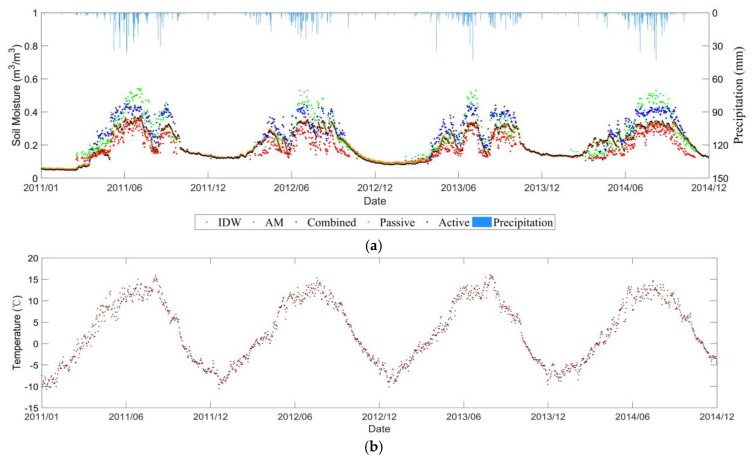
Temporal evolutions of (**a**) Climate Change Initiative (CCI) retrieved and *in-situ* measured soil moisture (**b**) temperature in the Central Tibetan Plateau Soil Moisture/Temperature Monitoring Network (CTP-SMTMN) network.

Figure 6 shows the evolution of the daily averaged soil moisture as well as temperature from 1 January 2011 to 31 December 2014 in the CTP-SMTMN network. In general, the soil moisture in the Tibet Plateau area shows significant annual and seasonal variation. From October to April of each year, when the soil moisture was less than 0.1 m^3^/m^3^, the corresponding soil temperature was below 0°. Thus, the low soil moisture values below 0.1 m^3^/m^3^ may be due to the soil freezing process, and the overall undulation is weak tending to be stable.

Indeed, the selected pixel is located in the northern part of Tibet, in the hinterland of the Qinghai-Tibet Plateau. Due to the influence of the summer monsoon in South Asia, precipitation is mainly concentrated from May to September. In addition, as the temperature rises, the surface frozen soil begins to melt, and the soil moisture value begins to gradually rise, reaching its peak in July and August. The temporal evolution of the retrieved soil moisture agreed with the extracted rainfall amount from TRMM data. The high precipitation amount led to a rapid increase in soil moisture. Then the soil moisture decreased due to the evapotranspiration and runoff. After September, as the precipitation decreased, the soil moisture began to decline, and the changes during the year were unimodal.

#### 3.1.2. Product Comparison Analysis

As can be seen from Figure 6, the three products of ESA CCI SM show similar changes to the station data, which captured the trend of ground measured soil moisture well. Among them, the value of the combined product is most consistent with the *in-situ* soil moisture. The values of the passive and active products are higher than the *in-situ* measurements, and the large fluctuations are in agreement with previous results [33].

Table 1 also reflects this fact very well. The ubRMSE of the combined product is 0.034, which is smaller than 0.066 for passive products and 0.039 for active products. Bias for the combined product is also the smallest among the three. Overall, the combined product has the highest accuracy, indicating that the integration of active and passive data sets in the Qinghai-Tibet Plateau has positive effects.

### 3.2. REMEDHUS Network 

**Figure 7:**
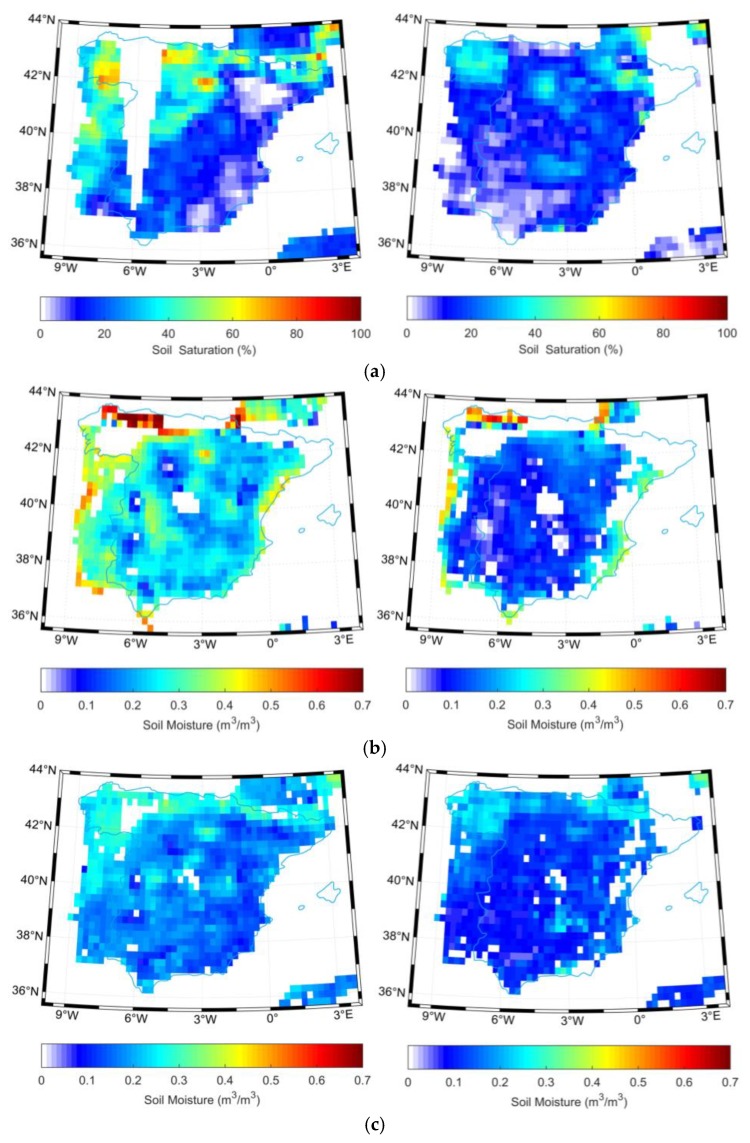
Extracted soil moisture retrieved from (**a**) active (**b**) passive (**c**) combined microwave data over Spain on 15 April (left) and 27 July (right) 2014.

**Figure 8:**
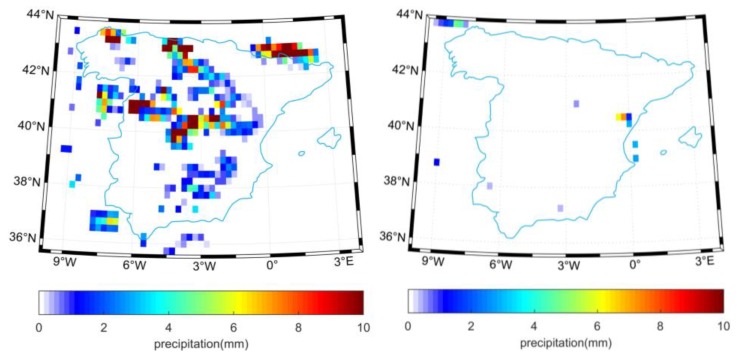
TRMM precipitation over Spain on 15 April (**left**) and 27 July (**right**) 2014.

Similarly, Figure 7 shows the CCI soil moisture in Spain for the two representative days corresponding to low and high vegetation amount, respectively. We can notice the higher soil moisture on 15 April than 27 July 2014 for the active, passive and combined products. Most of the pixels in the TRMM precipitation amount were missed in Figure 8, but a number of available pixels on 15 April are characterized by relative higher rainfall. However, on 27 July, almost all the pixels in the TRMM precipitation product were not retrievable. 

#### 3.2.1. Characteristics of Soil Moisture Evolution over Time

**Figure 9:**
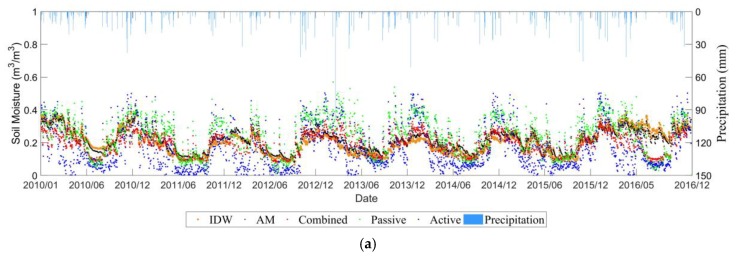

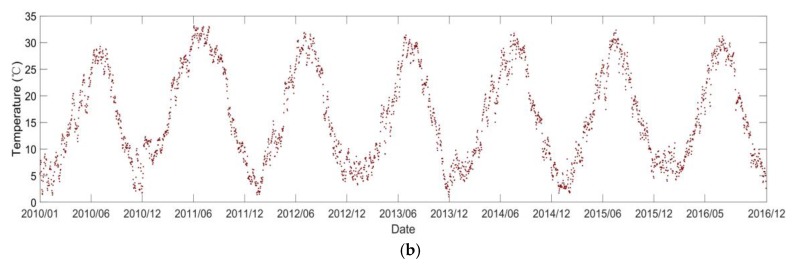
Temporal evolutions of (**a**) CCI retrieved and *in-situ* measured soil moisture (**b**) temperature in the REMEDHUS network.

Figure 9 illustrates the evolution of daily mean soil moisture over time from 0–5 cm depth as well as temperature from 1 January 2010 to 31 December 2016 in the REMEDHUS network. Overall, soil moisture in the REMEDHUS region also showed significant annual and seasonal changes. The REMEDHUS region is a semi-arid Mediterranean continental climate characterized by dry and warm summers and mild and humid winters. It can also be seen that the soil moisture value is lower from June to September each year (except for the *in-situ* value in 2016). From October to May of the next year, as the precipitation changes, the soil moisture value also fluctuates.

#### 3.2.2. Product Comparison Analysis

As can be seen from Figure 9, all satellite datasets captured well the evolution of the *in-situ* soil moisture cycles, although they had a larger dynamic range than the *in-situ* time series. Among them, the value of the combined product is most consistent with the value of the *in-situ* station, while passive product showed overestimation, and active product showed a certain underestimation with respect to the *in-situ* observations.

In Table 1, the combined product possessed an ubRMSE of 0.050 m^3^/m^3^, RMSE of 0.054, R of 0.710, and bias is −0.021 m^3^/m^3^, which is outperformed than the active-only and passive-only approaches. Thus, the combined product obtained the highest accuracy, and is most suitable in the REMEDHUS network, in agreement with the results in [28].

### 3.3. RISMA Network 

Over the RISMA site of Canada, several field campaigns including the Soil Moisture Active Passive Validation Experiments in 2012 and 2016 (SMAPVEX12, SMAPVEX16) were conducted for calibrating and validating the SMOS and SMAP soil moisture retrieval algorithms [50,51]. However, over the study site of Carman, the accuracy of soil moisture retrieval is not satisfactory due to the complex influences of vegetation and soil texture on the emissivity and backscattering [51]. In the current study, we used the RISMA soil moisture network to evaluate the performance of the ESA CCI soil moisture retrieval over the Carman site in Manitoba and another site in Saskatchewan. Unlike the previous two networks, the TRMM precipitation retrievals were not accessible here. Consequently, the precipitation amount information collected by the *in-situ* stations of the RISMA network was used to interpret the temporal variability of soil moisture.

**Figure 10:**
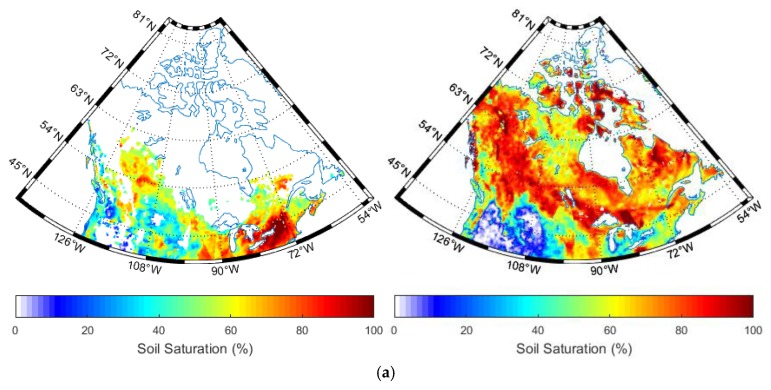

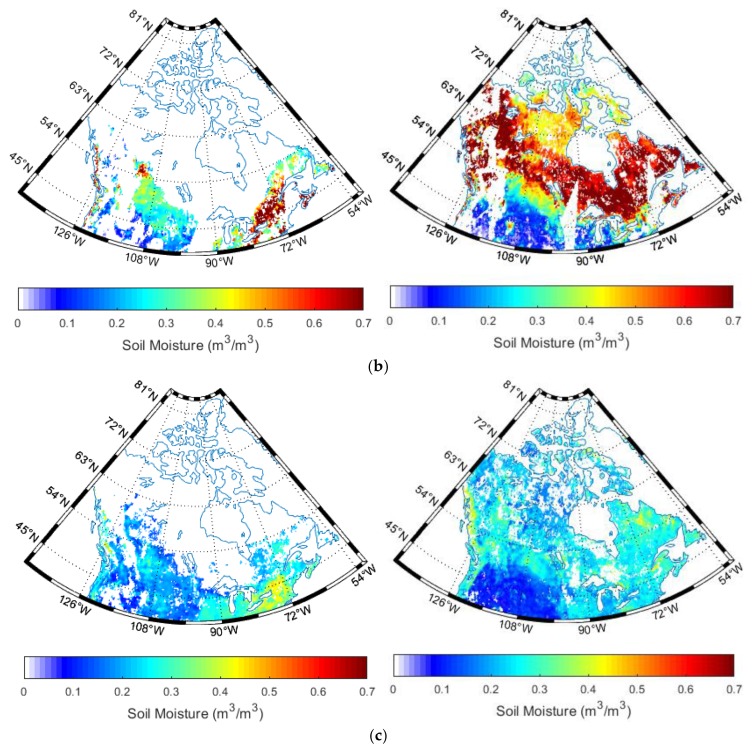
Extracted soil moisture retrieved from (**a**) active (**b**) passive (**c**) combined microwave data over Canada on 15 April (left) and 27 July (right) 2014.

In Figure 10, the retrieved soil moisture values from combined approach are lower than those from the active or passive approaches, in accordance with the previous two networks. Nevertheless, due to the high altitude and low temperature in Canada, the soils in April were often frozen over some of the study area. We can see that the CCI soil moisture on 15 April 2014 was characterized by around 85% invalid pixels. These invalid pixels are due to several reasons: (1) validity of the radiometer brightness temperature and the radar backscattering coefficients; (2) influence of soil thaw/freezing cycle; (3) the non-convergence of the radiative transfer models used for soil moisture retrievals; (4) the potential influence of radio frequency interference (RFI). However, we assumed that the RFI was not prominent in the Canada site, as the CCI retrieval rate of soil moisture in July is significantly higher than in April. In July, the rainfall became significant, leading to the increase in soil moisture. 

#### 3.3.1. Characteristics of Soil Moisture Evolution over Time

**Figure 11:**
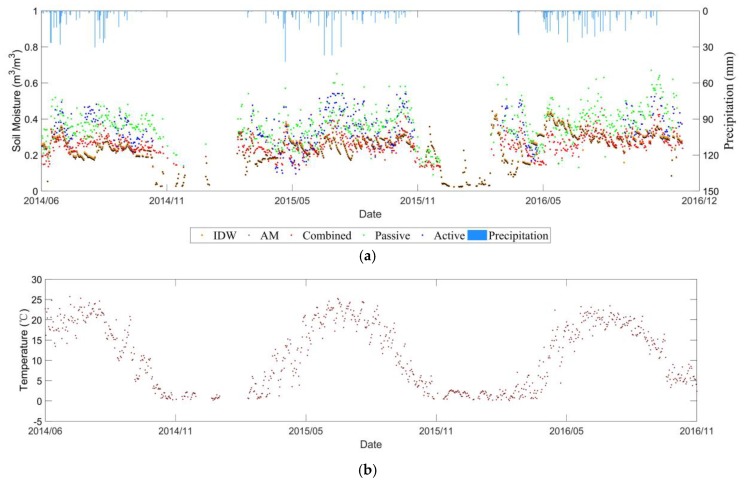
Temporal evolutions of (**a**) CCI retrieved and *in-situ* measured soil moisture (**b**) temperature over Manitoba site in the RISMA network.

**Figure 12:**
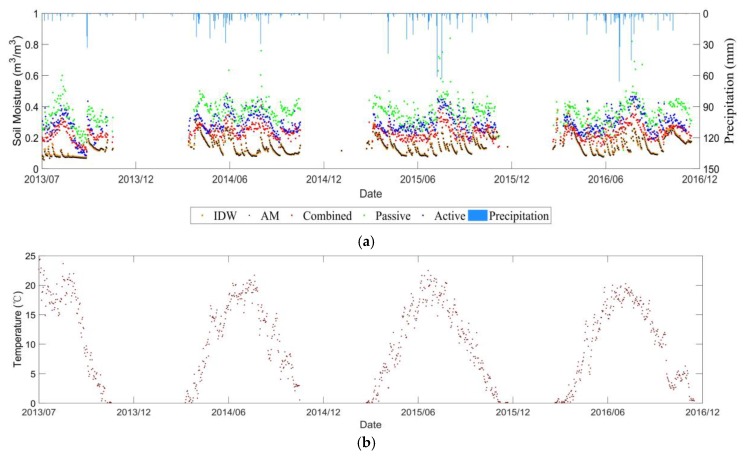
Temporal evolutions of (**a**) CCI retrieved and *in-situ* measured soil moisture (**b**) temperature over Saskatchewan site in the RISMA network.

Figure 11 and Figure 12 show the temporal evolution of daily average soil moisture at 0–5 cm depth as well as temperature from 1 June 2014 to 1 December 2016 in Manitoba, and from 1 July 2013 to 31 December 2016 in Saskatchewan. Due to the freezing of winter soils in Canada, there were almost no *in-situ* values in the winter over the *in-situ* stations, and the satellite signal cannot retrieve the soil moisture as well. These factors resulted in the lack of winter data for the comparison. In general, the soil moisture of the RISMA network does not have large fluctuations throughout the year. Following the precipitation events, the soil moisture values increased and decreased correspondingly.

#### 3.3.2. Product Comparison Analysis

For the RISMA network, a pronouncing bias was observed in the three products of ESA CCI SM. The soil moisture over Manitoba and Saskatchewan sites were significantly overestimated in all the three products. Nevertheless, similar temporal trends between the ESA CCI SM and the *in-situ* stations were captured, if the bias was compensated. The combined soil moisture product is the most consistent with the *in-situ* station. It can be further proved by the statistical metrics in Table 1 that the accuracies of the combined product over Manitoba and Saskatchewan sites are the highest. 

### 3.4. Different Upscaling Effects 

Based on the three soil moisture networks, our study shows similar effects between the simple AM and IDW methods to project the *in-situ* soil moisture within a pixel into the CCI SM spatial scale (see Figure 6a, Figure 9a, Figure 11a and Figure 12a). Table 1 and Table 2 compares the statistical metrics between the AM and IDW methods applied to the soil moisture ground stations. The weak difference is mainly due to the small number of available *in-situ* stations within a pixel of CCI soil moisture. In addition, the heterogeneity of soil texture, vegetation type and growth stages were not considered in the current upscaling process. As the spatial resolution of the CCI SM is around 0.25° × 0.25°, we assumed that the heterogeneities of soil texture, topography, land cover and meteorology were averaged within that pixel at coarse resolution. Furthermore, for the selected pixel, the temporal variability of the microwave signals was assumed to be dominated by the soil moisture, as the soil texture, topography and land cover did not change significantly along time within the pixel.

## 4. Conclusions

The European Space Agency (ESA) Climate Change Initiative (CCI) contributed to long-term soil moisture products which may benefit the study of global climate and hydrology. However, the quantitative validations under different climatic conditions are a prerequisite to understand their accuracies at different soil and vegetation conditions. Within this context, our study evaluated the performances of the retrieved soil moisture from active-only, passive-only and the combined approaches, using the ground soil moisture networks located in China, Spain and Canada. The selected three test sites were characterized by significant differences in vegetation and soil characteristics, providing diverse baselines for evaluations. 

Simple Arithmetic Mean (AM) and Inverse Distance Weighting (IDW) interpolation were used to upscale the ground soil moisture, for matching to the CCI retrieved soil moisture at coarse spatial resolution. Nevertheless, due to the limited number of ground measurements, we did not find significant differences between the AM and IDW methods applied for the aggregation of the ground measurements. Over the networks in China and Spain, the CCI retrieved soil moisture captured the variations of *in-situ* soil moisture, and positively responded to the rainfall events. However, over the RISMA network in Canada, a significant overestimation was observed for the three soil moisture products from active-, passive- and the combination, although the relatively temporal variation trends still follow similar pattern. Actually, a similar low performance of the SMAP soil moisture retrieval was reported over the Carman site [51], and this may due to the high heterogeneity in soil texture which required further intensive investigations. 

Over the three test sites, it is consistent that the soil moisture from the combined approach outperformed than those from active-only and passive-only approaches. The former obtained ubRMSE of 0.034, 0.050 and 0.050–0.054 m^3^/m^3^ over the test sites in China, Spain and Canada. Thus, we suggested using the combined soil moisture products for the climatic and hydrological studies. However, in our study, uncertainty may occur due to the simple upscaling approaches of AM and IDW. Therefore, in the forthcoming study, we shall investigate the alternative upscaling approaches such as kriging interpolation and Bayesian maximum entropy to obtain more representative *in-situ* soil moisture for the CCI pixels. 

## Figures and Tables

**Table 1:** The unbiased root mean square error (ubRMSE), root mean square error (RMSE), Pearson correlation coefficient (R) and bias of the comparison between the *in-situ* SM (AM) with the active (A), passive (P) and combined (C) CCI SM product for CTP-SMTMN, REMEDHUS and RISMA (Manitoba, Saskatchewan). Bold numbers indicate that the combined products perform best.

Networks	ubRMSE			RMSE			R			Bias		
	C	P	A	C	P	A	C	P	A	C	P	A
CTP-SMTMN	**0.034**	0.066	0.039	**0.059**	0.093	0.074	0.856	0.831	0.865	**−0.049**	0.066	0.062
REMEDHUS	**0.050**	0.082	0.103	**0.054**	0.088	0.115	**0.710**	0.693	0.612	**−0.021**	0.033	−0.049
Manitoba	**0.054**	0.076	0.093	**0.060**	0.165	0.145	0.420	0.449	0.330	**0.026**	0.146	0.111
Saskatchewan	**0.050**	0.072	0.065	**0.107**	0.233	0.161	**0.559**	0.507	0.474	**0.094**	0.222	0.148

**Table 2:** The ubRMSE, RMSE, R and bias of the comparison between the *in-situ* SM (IDW) with the active (A), passive (P) and combined (C) CCI SM product for CTP-SMTMN, REMEDHUS and RISMA (Manitoba, Saskatchewan). Bold numbers indicate that the combined products perform best.

Networks	ubRMSE			RMSE			R			Bias		
	C	P	A	C	P	A	C	P	A	C	P	A
CTP-SMTMN	**0.033**	0.067	0.039	**0.058**	0.095	0.075	0.858	0.830	0.866	**−0.048**	0.067	0.063
REMEDHUS	**0.065**	0.093	0.112	**0.065**	0.104	0.118	**0.583**	0.565	0.510	**−0.009**	0.045	−0.037
Manitoba	**0.055**	0.076	0.094	**0.059**	0.160	0.141	0.413	0.446	0.323	**0.021**	0.141	0.106
Saskatchewan	**0.052**	0.073	0.066	**0.105**	0.231	0.159	**0.544**	0.490	0.467	**0.091**	0.219	0.144
